# Effectiveness and Safety of First-Line Empirical Eradication Therapy with Rebamipide: Results from the European Registry on *Helicobacter pylori* Management (Hp-EuReg)

**DOI:** 10.3390/antibiotics15060531

**Published:** 2026-05-23

**Authors:** Dmitry S. Bordin, Dmitrii N. Andreev, Sayar R. Abdulkhakov, Irina N. Voynovan, Igor G. Bakulin, Natalia V. Bakulina, Ludmila G. Vologzhanina, Boris D. Starostin, Aiman S. Sarsenbayeva, Tatyana A. Ilchishina, Larisa V. Tarasova, Alla G. Kononova, Sergey A. Alekseenko, Oleg V. Zaytsev, Natalya N. Dekhnich, Pavel O. Bogomolov, Galina N. Tarasova, Olga A. Kolokolnikova, Igor B. Khlynov, Ekaterina Yu. Plotnikova, Natalia V. Baryshnikova, Maria A. Livzan, Marina F. Osipenko, Natalya V. Bakanova, Ekaterina V. Partsvania-Vinogradova, Margarita V. Chebotareva, Rustam A. Abdulkhakov, Inna V. Savilova, Anastasia G. Sushilova, Sergey V. Kolbasnikov, Alsu R. Khurmatullina, Igor V. Maev, Maria O. Tishchenko, Rimma E. Petrova, Ludmila V. Morkovkina, Yulia V. Tsyganova, Margarita D. Kozlova, Aleksei A. Iakovlev, Alexander S. Volkov, Elizaveta S. Moroz, Olga V. Gaus, Singla Alba, Oriol Farrés, Leticia Moreira, Pablo Parra, Olga P. Nyssen, Francis Megraud, Colm O’Morain, Javier P. Gisbert

**Affiliations:** 1 Department of Pancreatic, Biliary and Upper Digestive Tract Disorders, A. S. Loginov Moscow Clinical Scientific Center, 111123 Moscow, Russia; 2 Department of Internal Disease Propaedeutics and Gastroenterology, Russian University of Medicine, 127473 Moscow, Russia; 3 Department of Outpatient Therapy and Family Medicine, Tver State Medical University, 170100 Tver, Russia; 4 Department of Internal Diseases, Institute of Fundamental Medicine and Biology, Kazan (Volga Region) Federal University, 420008 Kazan, Russia; 5 Department of Hospital Therapy, Faculty of Medicine, Kazan State Medical University, 420012 Kazan, Russia; 6 Department of Propaedeutics of Internal Diseases, Gastroenterology and Dietetics named after S.M. Ryss, I.I. Mechnikov North-Western State Medical University, 191015 Saint Petersburg, Russia; 7 Department of Internal Medicine, Clinical Pharmacology and Nephrology, I.I. Mechnikov North-Western State Medical University, 191015 Saint Petersburg, Russia; 8 Department of Faculty Therapy No. 1, Perm State Medical University named after Academician E.A. Wagner, 614990 Perm, Russia; 9 Interdistrict Gastroenterological Center No. 1, City Polyclinic No. 38, 191015 Saint Petersburg, Russia; 10 Department of Therapy, Institute of Additional Professional Education, South Ural State Medical University, 454092 Chelyabinsk, Russia; 11 Medical Holding “SM-Clinic” (Medi Com LLC), 194355 Saint Petersburg, Russia; 12 Department of Hospital Therapy, I.N. Ulyanov Chuvash State University, 428015 Cheboksary, Russia; 13 Department of Hospital Therapy, Far Eastern State Medical University, 680000 Khabarovsk, Russia; 14 First Clinical Medical Centre, 601900 Kovrov, Russia; 15 Department of Faculty Therapy, Smolensk State Medical University, 214019 Smolensk, Russia; 16 Private Medical Center “Universal” LLC, 109382 Moscow, Russia; 17 Hepatology Department, M.F. Vladimirsky Moscow Regional Clinical Research Institute, 129110 Moscow, Russia; 18 Targeted Therapy Center LLC, 125008 Moscow, Russia; 19 Department of Internal Diseases Propaedeutics, Rostov State Medical University, 344022 Rostov-on-Don, Russia; 20 Department of Gastroenterology and Genetically Engineered Biological Therapy, MEDSI Clinical Hospital No. 1 in Otradnoye, 143442 Moscow Region, Russia; 21 Department of Faculty Therapy and Geriatrics, Ural State Medical University, 620028 Yekaterinburg, Russia; 22 Department of Outpatient Therapy, Postgraduate Education and Nursing Care, Kemerovo State Medical University, 650029 Kemerovo, Russia; 23 Department of Internal Diseases, Saint Petersburg State Pediatric Medical University, 194100 Saint Petersburg, Russia; 24 Department of Molecular Microbiology, Institute of Experimental Medicine, 197376 Saint Petersburg, Russia; 25 Department of Faculty Therapy and Gastroenterology, Omsk State Medical University, 644099 Omsk, Russia; 26 Department of Propaedeutics of Internal Diseases, Novosibirsk State Medical University, 630091 Novosibirsk, Russia; 27 Gastroenterology Service, Medical Center “Medicea”, 426000 Izhevsk, Russia; 28 N.V. Sklifosovsky Institute of Clinical Medicine, I.M. Sechenov First Moscow State Medical University, 119991 Moscow, Russia; 29 Gastrointestinal Oncology, Endoscopy and Surgery (GOES) Research Group, Althaia Xarxa Assistencial Universitària de Manresa, Institut de Recerca i Innovació en Ciències de la Vida i de la Salut a la Catalunya Central (IRIS-CC), 08243 Manresa, Spain; 30 Gastroenterology Service, Institute of Digestive and Metabolic Diseases (ICMDM), Hospital Clínic de Barcelona, CIBEREHD, IDIBAPS, University of Barcelona, 08036 Barcelona, Spain; 31 Department of Gastroenterology, Hospital Universitario de La Princesa, Instituto de Investigación Sanitaria Princesa (IIS-Princesa), Universidad Autónoma de Madrid (UAM) and Centro de Investigación Biomédica en Red de Enfermedades Hepáticas y Digestivas (CIBERehd), 28006 Madrid, Spain; 32 INSERM U1312 BRIC, University of Bordeaux, 33076 Bordeaux, France; 33 School of Medicine, Trinity College Dublin, D02 PN40 Dublin, Ireland

**Keywords:** *Helicobacter pylori*, eradication, antimicrobial therapy, rebamipide, treatment, registry

## Abstract

Background/Objective: Eradication of *Helicobacter pylori* typically uses acid-suppressing agents plus antimicrobials, but treatment failure may reach 20–30%. Adjunctive rebamipide has been proposed as a strategy to improve treatment outcomes. This study aimed to assess the effectiveness and safety of first-line empirical therapy when rebamipide was added to eradication regimens. Methods: Treatment-naïve patient data were collected between January 2020 and August 2024 from the European Registry on Helicobacter pylori Management (Hp-EuReg), an international, prospective, multicentre, observational study. Rebamipide-containing regimens were analysed and compared with those without rebamipide. Effectiveness was assessed using a modified intention-to-treat approach. Results: Overall, 4581 patients (mean age 48 ± 15 years, 64% women) from 15 Russian cities were included. From those, 820 patients received eradication therapy with rebamipide and 3761 without rebamipide. Overall effectiveness was significantly higher with rebamipide (731/763, 96%) than without it (2785/3059, 91%) (*p* = 0.0002). Rebamipide-containing triple therapy consisting of a proton-pump inhibitor (PPI), clarithromycin and amoxicillin was associated with effectiveness above 90%, while rebamipide-containing quadruple therapy consisting of a PPI, clarithromycin, amoxicillin and bismuth yielded a 97% eradication rate, compared with 79% and 95%, respectively, without rebamipide (*p* < 0.001). Adverse events were significantly more frequent in patients receiving rebamipide-containing regimens (46% vs. 29%, *p* < 0.0001), though none were serious or caused discontinuation. Compliance was similar in both groups (99% vs. 98.5%; *p* > 0.05). Conclusions: In this observational registry cohort, first-line empirical triple therapy with amoxicillin–clarithromycin, and quadruple therapy with amoxicillin–clarithromycin–bismuth, both prescribed with rebamipide, were associated with higher eradication rates than regimens without rebamipide.

## 1. Introduction

*Helicobacter pylori (H. pylori)* is a microaerophilic, spiral-shaped, gram-negative bacterium that colonizes the human gastric mucosa and is a leading cause of numerous gastroduodenal diseases, including chronic gastritis, gastroduodenal ulcer, MALT lymphoma and gastric adenocarcinoma [1,2]. The global infection rate of *H. pylori* between 2011 and 2022 was estimated at 43% [3].

Eradication therapy, which combines acid-suppressing agents—proton pump inhibitors (PPIs) or potassium-competitive acid blockers (P-CABs)—with antimicrobial drugs—clarithromycin (C), amoxicillin (A), metronidazole (M), tetracycline (Tc)—can resolve inflammatory changes in the gastric mucosa and prevent the development of precancerous conditions, such as atrophic gastritis and intestinal metaplasia [1,2,4,5]. An acceptable level of eradication therapy effectiveness is generally considered to be above 90% [6,7]. However, achieving this threshold using empirical regimens is currently challenging, representing a significant clinical problem for general practitioners and gastroenterologists worldwide [8,9].

Several factors contribute to the limited effectiveness of eradication therapy, particularly increasing *H. pylori* antibiotic resistance [9,10], while poor patient adherence remains another important determinant of treatment failure [9]. In many European regions, including Russia, clarithromycin resistance in *H. pylori* exceeds the 15% threshold above which empirical clarithromycin-containing triple therapy is generally discouraged [2,10]. The approaches of supplementing classical eradication therapy regimens with additional components are actively studied to increase effectiveness and improve adherence, often by reducing the frequency of adverse events (AEs) [11,12]. Convincing evidence from large meta-analyses and prospective multicentre controlled trials supports several approaches to improve eradication therapy effectiveness, including the addition of bismuth salts, the use of PPIs with minimal metabolism dependence on *CYP2C19* polymorphism, doubling the standard daily PPI dosage, and the adjunct use of probiotics [11,13,14,15].

Among these strategies, the addition of rebamipide, widely used in Japan, South Korea and Russia as a gastro- and entero-protective agent, has emerged as a promising approach to optimize eradication therapy effectiveness [16,17]. Developed and introduced into clinical practice in Japan in 1990, rebamipide is now registered and used in over 10 countries worldwide [16,17]. Rebamipide does not have a direct anti-*H. pylori* effect; however, experimental studies have shown that it inhibits *H. pylori* adhesion to epithelial cells of the gastric mucosa and reduces *H. pylori*-induced NF-kB activation and IL-8 production [18,19]. Two independent meta-analyses of randomized controlled trials (RCTs) conducted in different populations demonstrated that including rebamipide in eradication therapy significantly increases overall treatment effectiveness; and specifically, across varying therapeutic dual, triple and quadruple bismuth-containing regimens [20,21].

This study addressed the need for real-world clinical data to complement and contextualize findings from RCTs conducted in selected patient populations. This study therefore aimed to assess the effectiveness and safety of adding rebamipide to first-line empirical antimicrobial eradication therapy in the context of increasing *H. pylori* antibiotic resistance, particularly to clarithromycin.

## 2. Methods

### 2.1. European Registry on H. pylori Management (Hp-EuReg)

Hp-EuReg is an international, multicentre, prospective, non-interventionist registry promoted by the European Helicobacter and Microbiota Study Group (www.helicobacter.org) which has been recording information on the management of *H. pylori* infection since 2013. The present analysis was observational and non-randomized.

The Hp-EuReg protocol [22] establishes national coordinators in each of the participating countries, where selected gastroenterologists enter data into the registry. All the investigators are gastroenterologists managing patients infected with *H. pylori* and working at centres with a valid confirmatory testing method. For all patients included in the registry, active *H. pylori* infection had to be documented before treatment using at least one validated diagnostic method available at the participating centre, including the 13C-urea breath test, *H. pylori* stool antigen test, histology, rapid urease test and/or culture. Records without a documented positive baseline *H. pylori* diagnosis were not eligible for the present analysis.

The study complies with the last revision of ethical guidelines of the Declaration of Helsinki [23] as reflected in the prior approval granted by the institution’s human research committee as well as with the guidelines for good clinical practice [24]. The study was classified by the Spanish Agency for Medicines and Health Products and is registered in ClinicalTrials.gov (NCT02328131). It was approved by the ethics committee of La Princesa University Hospital.

The World Registry on *H. pylori* Management (WorldHpReg) intellectual property was registered on 24 March 2024, under the Spanish Intellectual Property Office (registration number M-001958/2024; asiento registral 16/2024/3986). Exploitation rights are shared among the principal investigator and the Scientific Directors. In January 2026, WorldHpReg was included in the Heads of Medicines Agencies–European Medicines Agency Catalogue of real-world data sources (EU PAS number: EUPAS1000000912), enabling its use within European regulatory and research frameworks.

### 2.2. Participants

The registry allows the evaluation of the results of *H. pylori* treatment in real clinical practice by European gastroenterologists from 37 countries. Hp-EuReg operates under the guidance of the Scientific Committee, providing quality control and scientific integrity of the data and articles written.

Variables and outcomes were recorded using an electronic case report form (e-CRF) provided by the collaborative research platform Research Electronic Data Capture (REDCap) [25] hosted at “Asociación Española de Gastroenterología” (AEG; www.aegastro.es), a non-profit scientific and medical society focused on gastroenterology research. Data were anonymised and the following variables were recorded: patients’ demographics, any previous eradication attempts, treatments used, and effectiveness and safety outcomes. Previous eradication attempts were captured in structured e-CRF fields, including treatment line and prior eradication regimens. During data cleaning, these variables were cross-checked with the recorded treatment line; for the present first-line empirical treatment analysis, patients with previous eradication attempts were excluded from the first-line effectiveness population or classified as subsequent treatment lines, as appropriate. Further information on the variables is available in the published protocol [22]. Written informed consent was obtained from all patients prior to study entry.

### 2.3. Data Management

For the present study, data were extracted and subsequently analysed from 15 Russian cities, each contributing more than 100 patients to the Hp-EuReg: Moscow, Saint Petersburg, Kazan, Cheboksary, Chelyabinsk, Izhevsk, Novosibirsk, Khabarovsk, Omsk, Perm, Kovrov, Smolensk, Rostov-on-Don, Krasnoyarsk, and Tver. The data included in this study were collected from January 2020 to August 2024. Although the Hp-EuReg has been recording data on the management of H. pylori infection since 2013, rebamipide-specific treatment data from Russian centres have been available in the registry since 2020; therefore, this period was selected for the present analysis.

All Russian centres actively contributing data to the Hp-EuReg during the study period and meeting the predefined contribution threshold were eligible for inclusion; centres were not selected according to treatment outcomes or rebamipide prescribing patterns. As rebamipide is currently available in Europe only in Russia, Russian centres were the only Hp-EuReg sites in which its use as an adjunct to *H. pylori* eradication therapy could be evaluated.

The authors conducted a quality control check of all records included for each city and centre. After extracting the data and prior to the statistical analysis, the database was reviewed for inconsistencies and subsequent data cleaning. The data quality review process evaluated whether the study selection criteria had been met and whether data were correctly collected, in order to ensure that the study was conducted according to the highest scientific and ethical standards. Data discordances were resolved by consulting the investigators and through group emailing.

Records corresponding to eradication therapy regimens containing rebamipide, used in addition to antimicrobial agents, were analysed and compared with those not containing rebamipide, in order to assess effectiveness, safety and treatment compliance.

### 2.4. Statistical Analysis

#### 2.4.1. Categorisation and Definition of Variables

To enhance data interpretation, variables were systematically categorised. The different dosage schedules of PPIs—including omeprazole, lansoprazole, pantoprazole, rabeprazole, and esomeprazole—were standardised based on their relative potency in omeprazole equivalents. PPI doses were further classified into three categories as described by Graham et al. and Kirchheiner et al. [26,27]: low-dose (4.5–27 mg of omeprazole equivalents given twice a day), standard-dose (32–40 mg of omeprazole equivalents given twice a day), and high-dose (54–128 mg of omeprazole equivalents given twice a day). This classification was labelled as “PPI dose” to facilitate the analysis.

The duration of treatment, referred to as “length,” was categorized into 7, 10, or 14 days, as these were the most frequently prescribed regimens.

To evaluate the effectiveness of different *H. pylori* eradication regimens, first-line empirical eradication treatments were classified into four groups: a triple regimen combining a PPI with clarithromycin and amoxicillin (PPI + C + A); and three bismuth-based quadruple regimens: combining a PPI with clarithromycin, amoxicillin, and bismuth (PPI + C + A + B); combining a PPI with amoxicillin, josamycin and bismuth (PPI + A + J + B), and combining a PPI with metronidazole, tetracycline, and bismuth (PPI + M + Tc + B).

Effectiveness was assessed using three sets of patients as follows: (1) the intention-to-treat (ITT) analysis included all patients registered up to 6 months prior to the data extraction date, where cases lost to follow-up were considered treatment failures; (2) a modified intention-to-treat (mITT) analysis included all cases registered up to the data extraction date who had completed the follow-up (a confirmatory test—success or failure—was available after the eradication treatment), and regardless of compliance; and (3) the per-protocol (PP) analysis included all patients with completed follow-up who had taken at least 90% of the treatment drugs as defined in the protocol [22]. This study focused on the mITT analysis, as it was designed to most closely reflect real-world clinical practice.

Only patients who were empirically treated, meaning those who did not receive a susceptibility-guided antibiotic prescription, were included in the analysis.

*H. pylori* eradication was evaluated at least four weeks after completing the treatment by at least one of the following diagnostic methods: 13C-urea breath test, *H. pylori* stool antigen test, histology, rapid urease test and/or culture.

The incidence of AEs and compliance rate were assessed by interviewing patients both at the appointment and using a pre-arranged questionnaire. Adherence to treatment, defined as compliance, was evaluated through face-to-face interviews using open-ended questions and a structured questionnaire. Patients who had taken at least 90% of their prescribed drugs were considered compliant, while those with lower adherence were considered non-compliant.

#### 2.4.2. Data Analysis

The distribution of continuous variables was assessed using the Shapiro-Wilk test and visual inspection of histograms and Q-Q plots. The mean value and standard deviation (SD) were used to describe quantitative indicators with an approximately normal distribution. Median and interquartile range were used to describe the distribution of data that were not normally distributed. Between-group comparisons for continuous variables were performed using parametric or non-parametric tests, as appropriate. Statistical significance of categorical variables was determined using Pearson’s chi-square test or Fisher’s exact test. Confidence intervals (CIs) were calculated based on Fisher’s angular transformation. Differences were considered statistically significant at *p* < 0.05.

Multivariate analysis was performed using a logistic regression model by the stepwise likelihood method. The eradication rate in the mITT population was set as the dependent variable, and the independent variables were the following: sex (female [reference category] vs. male), age, treatment duration (7 [reference category] vs. 10 or 14 days), dose of PPI (low [reference category], vs. standard or high), compliance (yes: ≥90% of drug intake [reference category] vs. no: <90%), use of rebamipide (yes [reference category] vs. no), and the prescribed first-line eradication treatment separately, using the categories previously described above (PPI + C + A [reference category] vs. PPI + M + Tc + B vs. PPI + C + A + B vs. PPI + A + J + B vs. others –encompassing the remaining prescriptions in Russia). Likewise, safety was evaluated. In this model, the incidence of at least one AE was set as the dependent variable, and the same independent variables as for the effectiveness model were included.

The odds ratio (OR) and 95% CI for eradication in each variable were calculated relative to a reference category; and therefore, the OR was considered treatment success within each independent variable studied when associated with a higher mITT rate, at a *p*-value of <0.05.

## 3. Results

### 3.1. Baseline Characteristics of the Population

A total of 4581 patients (mean age 48 ± 15 years, 64% women) were included in the analysis: 820 patients received eradication therapy with rebamipide (mean age 47 ± 15 years, 60% women), and 3761 patients received eradication therapy without rebamipide (mean age 49 ± 15 years, 65% women) (Table 1). The main indications for eradication therapy in the overall group of patients were dyspepsia (66%) and peptic ulcer disease (18%). Baseline characteristics differed between groups: patients receiving rebamipide were more frequently male and had a higher prevalence of peptic ulcer disease, particularly gastric ulcer disease, whereas dyspepsia was more frequent in the non-rebamipide group (Table 1). This imbalance suggests that rebamipide was more frequently prescribed to patients with organic mucosal lesions and, therefore, with a potentially more compromised gastric mucosa.

Among the 820 patients receiving regimens with rebamipide, 789 received a first-line therapy, while the remaining patients received a second-line (n = 27), third-line (n = 3), and fourth-line (n = 1) therapy.

The most frequently prescribed rebamipide-containing regimens were PPI + C + A + B (n = 486) and PPI + C + A (n = 168) (Table 1). Rebamipide was prescribed at a dose of 100 mg three times a day, regardless of meal intake, for the entire duration of eradication therapy.

### 3.2. Effectiveness

**Figure 1:**
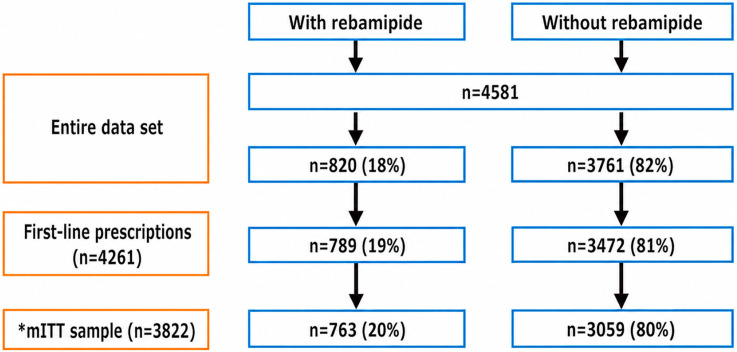
Flow-Chart of the study. *mITT (modified ITT)—includes all patients who completed follow-up observation (i.e., who underwent a confirmatory test after eradication therapy), regardless of adherence to protocol drug intake.

The effectiveness analysis included 4261 patients who received first-line eradication therapy, comprising 789 (19%) regimens with rebamipide and 3472 (81%) without (comparison group). The mITT effectiveness was assessed in 763 (20%) patients receiving rebamipide and 3059 (80%) patients without rebamipide (Figure 1). Patients excluded from the mITT analysis were those without a valid post-treatment eradication confirmation test, including patients lost to follow-up or not tested according to the registry protocol.

Regimens containing rebamipide were associated with significantly higher overall effectiveness (96% vs. 92%; *p* < 0.001), regardless of treatment duration or PPI dose (Table 2). However, rebamipide prescriptions administered with either high- or standard-dose PPI regimens achieved the best eradication outcomes (99% vs. 95%; *p* < 0.05; and 95% vs. 91%; *p* < 0.001, respectively). Low-dose PPI prescriptions did not differ significantly, regardless of the addition of rebamipide.

Rebamipide-containing 14-day regimens were associated with significantly higher effectiveness (96% vs. 91%; *p* < 0.001). Shorter 7-day prescriptions showed suboptimal overall effectiveness without rebamipide (80%). Outcomes in the rebamipide group could not be evaluated owing to the limited number of patients treated for 7 days (n = 20). The addition of rebamipide to a 10-day regimen did not significantly enhance outcomes, raising effectiveness from 93% to 96% (*p* = 1.0) (Table 2).

A significant association with rebamipide use was observed across different treatment combinations, particularly in PPI + C + A (92% vs. 79%, *p* < 0.001), regardless of the PPI dose. When considering all treatment durations, the overall effectiveness with PPI + C + A was 94% in the rebamipide group and 79% in the non-users (*p* < 0.001). When analysing 14-day PPI + C + A prescriptions, the eradication rate reached 93% when rebamipide was added to the regimen compared with 78% in the non-users (*p* < 0.001). For quadruple regimens, rebamipide use reported an overall increase in the eradication rate from 95% to 97% with PPI + C + A + B therapy, from 92% to 100% with PPI + A + B + J, and 94% to 100% with PPI + M + Tc + B (Table 2). However, statistical differences were shown only for the PPI + C + A + B group (*p* < 0.05).

The influence of PPI dosage on the effectiveness of first-line *H. pylori* eradication regimens, with and without rebamipide, is presented in Table 2, stratified by specific treatment combinations. Across nearly all PPI dose levels and regimens, the addition of rebamipide was associated with higher mITT eradication rates. The most striking difference was observed in the PPI + C + A regimen, where the overall eradication rate was significantly higher with rebamipide than without (92.3% vs. 78.6%). Within this regimen, the benefit of rebamipide was most pronounced at standard PPI doses (91.7% vs. 76.1%), while both groups achieved 100% eradication at high doses, albeit in a small sample. For the PPI + C + A + B regimen, which already demonstrated high efficacy without rebamipide (94.6%), rebamipide use was associated with higher eradication rates to 97% overall, with perfect 100% efficacy observed in the high-dose PPI subgroup.

The multivariate analysis of effectiveness identified independent factors associated with higher mITT eradication rates, including compliance (OR 3.68, 95% CI 1.82–7.42), high-dose PPIs (OR 1.99, 95% CI 1.19–3.33), and the use of rebamipide (OR 2.35, 95% CI 1.59–3.47). Specific regimens also remained associated with higher eradication rates after adjustment for rebamipide use and the other independent factors; notably, PPI + C + A + B (OR 4.21, 95% CI 3.10–5.72), PPI + M + Tc + B (OR 3.55, 95% CI 1.95–6.46), and PPI + A + J + B (OR 2.86, 95% CI 1.2–4.76).

Further multivariate analyses were conducted according to treatment regimen. Separate models were fitted for the PPI + C + A and PPI + C + A + B regimens, given the adequate sample size of both rebamipide and non-rebamipide users within these groups. In contrast, multivariate analyses for the PPI + M + Tc + B and PPI + A + J + B regimens were not conducted because most patients were treated without rebamipide, limiting meaningful statistical evaluation of this variable.

In both the PPI + C + A and PPI + C + A + B models, treatment duration was included only for the 10- and 14-day categories, as 7-day regimens comprised a negligible number of cases. Additionally, the model evaluating PPI + C + A therapy was restricted to standard-dose PPIs due to similar sample size limitations.

For triple therapy, the factors independently associated with higher effectiveness were treatment compliance (OR: 18.7, 95% CI: 1.92–181.8) and rebamipide supplementation (OR: 3.52, 95% CI: 1.59–7.81). In the PPI + C + A + B cohort, variables independently associated with increased treatment success were high-dose PPI use (OR: 2.75, 95% CI: 1.14–6.63) and rebamipide use (OR: 1.85, 95% CI: 1.03–3.33).

### 3.3. Safety and Compliance

The incidence of at least one AE was assessed in 789 patients receiving eradication therapy with rebamipide and in 3472 patients receiving eradication therapy without rebamipide. The overall incidence of AEs was significantly higher in patients treated with rebamipide-containing regimens compared with those receiving rebamipide-free regimens (46% vs. 29%, *p* < 0.0001). The type of AEs reported is presented in Table 3 and Table 4. Overall, patients in the rebamipide-free cohort experienced a higher incidence of diarrhoea (7.4% vs. 5.1%, *p* = 0.030). Conversely, the addition of rebamipide was associated with a markedly and significantly higher incidence of several gastrointestinal AEs, including nausea (14.4% vs. 6.8%, *p* < 0.001), vomiting (6.1% vs. 1.8%, *p* < 0.001), dyspepsia (13.3% vs. 5.5%, *p* < 0.001), and abdominal pain (6.1% vs. 2.8%, *p* < 0.001). The incidence rate of dysgeusia, heartburn, anorexia, and other AEs was comparable between the two groups. The broader differences in AEs were observed with the PPI + C + A regimen, where rebamipide use was associated with significantly higher rates of nausea (10.1% vs. 5.2%, *p* = 0.043), dyspepsia (10.1% vs. 3.7%, *p* = 0.003), and in the overall incidence of AEs reported with this triple therapy (34.9% vs. 19.5%, *p* < 0.001). A similar pattern was observed with PPI + C + A + B, with the rebamipide group showing higher incidence of nausea (13.4% vs. 4.9%, *p* < 0.001), vomiting (8.8% vs. 2.3%, *p* < 0.001), dyspepsia (20.5% vs. 6.4%, *p* < 0.001), and abdominal pain (8.3% vs. 2.9%, *p* < 0.001), and overall AEs (54.4% vs. 31.6%, *p* < 0.001). The severity of AEs in those rebamipide-containing regimens did not differ from those observed in the rebamipide-free group and were predominantly mild or moderate. None of the AEs led to treatment discontinuation.

The multivariate analysis of safety identified several factors that were independently associated with a reduced incidence of at least one AE overall, such as treatment compliance (OR: 0.14, 95% CI: 0.08–0.26) and male sex (OR: 0.70, 95% CI: 0.61–0.81). Similarly, standard-dose PPIs showed a protective association (OR: 0.45, 95% CI: 0.36–0.56). Conversely, other factors were associated with a higher incidence of reported AEs. Rebamipide use was associated with increased AE reporting (OR: 2.54, 95% CI: 2.13–3.03). Regarding the treatment regimens, both PPI + C + A + B (OR: 2.22, 95% CI: 1.78–2.77) and PPI + M + Tc + B (OR: 1.69, 95% CI: 1.19–2.41) were associated with a significantly higher incidence of experiencing at least one AE.

Specific multivariate analyses for AEs were conducted for the PPI + C + A and PPI + C + A + B regimens, including duration with 10- and 14-day lengths only as previously stated. In the model of PPI + C + A, prescribed with standard-dose PPIs, the factor significantly associated with a lower incidence rate of AEs was treatment compliance (OR: 0.13, 95% CI: 0.03–0.50). Conversely, rebamipide supplementation was associated with a higher incidence of AEs (OR: 3.83, 95% CI: 2.25–6.52).

In the PPI + C + A + B model, factors independently associated with a reduced likelihood of AEs were treatment compliance (OR: 0.16, 95% CI: 0.07–0.39), male sex (OR: 0.70, 95% CI: 0.59–0.84), and the use of standard-dose PPIs (OR: 0.49, 95% CI: 0.37–0.64). Conversely, rebamipide supplementation was associated with a higher incidence of AEs in this group (OR: 2.65, 95% CI: 2.13–3.29).

Additionally, serious AEs were reported in 5 of 978 patients (0.5%) in the control group, whereas none occurred in patients treated with rebamipide-containing regimens.

Overall compliance was similar between patients treated with rebamipide (99%) and those treated without it (98.5%). This trend of high and comparable compliance was consistently observed across the individual treatment regimens, including PPI + C + A (99% with rebamipide vs. 97% without) and PPI + C + A + B (99% with vs. 99% without). Although compliance in the PPI + M + Tc + B with-rebamipide group was lower (83%) compared to its without-rebamipide counterpart (98%), this finding is based on a very small sample size (n = 6) and is unlikely to be statistically reliable (Table 5).

## 4. Discussion

The present study, conducted using data from the Hp-EuReg provided by Russian centres, showed that rebamipide-containing regimens were associated with significantly higher overall eradication rates compared with regimens without rebamipide (96% vs. 92%; *p* < 0.001). These findings are consistent with the most recent meta-analysis summarizing six controlled studies from Russia, which also confirmed a significant increase in effectiveness with the addition of rebamipide [28]. The relatively high eradication rates achieved in regimens without rebamipide in our cohort likely reflect the frequent use in Russia of highly effective 14-day bismuth-based quadruple therapies (PPI + M + Tc + B and PPI + C + A + B), in line with evidence from meta-analyses [13,29].

In contrast, the effectiveness of first-line empirical PPI + C + A therapy was suboptimal (79%), consistent with the global decline in the effectiveness of this regimen due to rising antibiotic resistance. Similar rates have been reported in a recent meta-analysis of 80 RCTs (80%) [30] and in the Hp-EuReg across 27 European countries (81.5% in 21,533 patients) [31]. Importantly, in our study, rebamipide-containing PPI + C + A therapy was associated with eradication rates above the clinically significant 90% threshold. When combined with PPI + C + A + B therapy, rebamipide was associated with further improvement in outcomes, achieving a 97% eradication rate. These findings align with results from an RCT by Simanenkov et al. in 2017, which reported high effectiveness (95%) for PPI + C + A + B therapy containing rebamipide [32].

In certain subgroups with small sample sizes, such as PPI + A + J + B and PPI + M + Tc + B regimens, rebamipide was associated with eradication rates of 100%, compared with 92% and 94%, respectively, in the rebamipide-free groups. However, these results should be interpreted cautiously due to the limited number of patients receiving rebamipide in these specific regimens.

On multivariate analysis, treatment compliance and rebamipide supplementation were independently associated with higher eradication rates in triple PPI + C + A therapy. In the PPI + C + A + B regimen, high-dose PPI use and rebamipide supplementation were independently associated with improved effectiveness. These findings are consistent with several meta-analyses and observational studies highlighting the importance of PPI dosing, patient adherence, and adjunctive therapies in optimising *H. pylori* eradication outcomes [33,34,35].

The clinical relevance of these findings should also be considered in the context of clarithromycin resistance. Although individual-level antimicrobial susceptibility data were not available in the Hp-EuReg for the patients included in this analysis, recent Russian data indicate that clarithromycin resistance exceeds the 15% threshold in several regions [10]. Rebamipide has no direct anti-*H. pylori* activity, but its effects on mucosal protection and gastric mucosal microcirculation may theoretically improve local antibiotic delivery to the infected gastric epithelium. This mechanism could be particularly relevant in infections with low-level resistance or heteroresistant *H. pylori* populations, where higher local antibiotic exposure might contribute to eradication success.

The particularly high eradication rates observed when rebamipide was combined with high-dose PPI therapy may reflect complementary mechanisms. High-dose PPI therapy provides more potent and sustained acid suppression, which may improve the stability and antimicrobial activity of acid-labile antibiotics and create a more favourable environment for *H. pylori* eradication [36,37]. Rebamipide, in turn, may contribute through restoration of mucosal barrier integrity, anti-inflammatory effects, and improvement of gastric mucosal microcirculation, potentially facilitating antibiotic delivery to the infected mucosa [17]. However, this possible interaction should be interpreted as hypothesis-generating, since the present registry did not include intragastric pH monitoring, pharmacokinetic assessment, local antibiotic concentration measurements, or molecular evaluation of mucosal repair. Dedicated mechanistic and pharmacokinetic studies are needed to clarify whether rebamipide and high-dose PPI therapy exert true synergistic effects.

Regarding safety, the incidence of AEs was higher in the rebamipide-user groups compared with controls (46% vs. 29%). This difference should be interpreted cautiously because of the observational, non-randomised design and the baseline imbalance between groups. Many of the symptoms reported as AEs (dyspepsia, nausea, abdominal pain, etc.) may have been related to underlying gastrointestinal conditions rather than to the eradication regimen itself. Indeed, peptic ulcer disease was significantly more frequent among patients receiving rebamipide. Moreover, the Hp-EuReg records AEs at the treatment-line level and does not assign causality to individual drugs; therefore, the observed AEs may represent the cumulative burden of multidrug eradication therapy, including antibiotics, bismuth, and PPI, rather than a specific effect of rebamipide. The types of AEs reported were predominantly mild gastrointestinal symptoms and are consistent with known effects of both eradication regimens and background gastrointestinal disease. Importantly, the AEs reported were mild to moderate, did not lead to treatment discontinuation, and overall compliance remained very high (99% vs. 98.5%), suggesting that the AE profile was clinically manageable in routine practice. However, further controlled studies with formal attribution analysis are needed to disentangle drug-specific safety effects. This is consistent with previous evidence from the Korean registry of patients with gastroesophageal reflux disease and peptic ulcer disease, showing that rebamipide has a favorable safety profile without potential risk of serious AEs [38]. Earlier meta-analyses (published in 2014 and 2019) reported no difference in AEs incidence between regimens with and without rebamipide [20,21], while the most recent meta-analysis (2022) focusing on PPI + C + A therapy even suggested a protective effect of rebamipide, with a significant reduction in the AEs risk [28]. A recently published umbrella review (2026) on the use of rebamipide in several gastrointestinal diseases (including *H. pylori* infection) indicated a reduction in the incidence of AEs during eradication treatment [39].

The discrepancy between registry-based and trial-based safety findings may be explained by methodological differences. Randomisation in RCTs helps minimizing bias and provides a standardized assessment of the estimate of a drug effect, whereas registry studies reflect real-world clinical practice, where treatment choices may be influenced by patient vulnerability (e.g., advanced age or comorbidities). Registries therefore remain more prone to confounding when assessing AEs rates.

Our study has several limitations. First, the observational, non-randomised design precludes causal inference, and the results should be interpreted as associations rather than proof of a direct rebamipide effect. Second, rebamipide was prescribed at the physician’s discretion, which creates a risk of confounding by indication; patients receiving rebamipide had a higher prevalence of peptic ulcer disease, suggesting a clinically more compromised gastric mucosa. Although multivariable adjustment was performed, residual and unmeasured confounding cannot be excluded. Third, the study is based on registry data and includes heterogeneous diagnostic methods for confirming eradication success, which may limit direct comparisons between specific regimens, even though this heterogeneity likely affected both groups similarly. Fourth, compliance was assessed using patient self-report and physician-entered registry data, which may overestimate true adherence [40]. Fifth, the analysis was restricted to a single country (Russia), which may reduce the generalizability of the findings to other populations; however, this restriction reflects the fact that rebamipide is available in Europe only in Russia. Sixth, the registry did not include individual-level antimicrobial susceptibility, intragastric pH, pharmacokinetic, local antibiotic concentration, or molecular mucosal repair data, limiting mechanistic interpretation. Seventh, the early study period (2020–2021) overlapped with the COVID-19 pandemic, which temporarily disrupted elective endoscopy, outpatient visits, and follow-up logistics [41,42]. These disruptions may have introduced selection bias in the overall cohort and influenced the completeness of eradication confirmation. However, because the rebamipide and non-rebamipide groups were recruited contemporaneously from the same centres and subjected to the same pandemic-related constraints, a differential bias affecting the between-group comparison of effectiveness is unlikely. Nonetheless, this is the first large-scale study to evaluate rebamipide-containing regimens in real-world clinical practice within a context of increasing *H. pylori* antibiotic resistance, particularly to clarithromycin [10,43]. Our findings are consistent with prior meta-analyses from Japan, South Korea, and Russia [20,21,28], supporting the role of rebamipide as a potentially valuable add-on drug in eradication therapy. Strengths of this study include its prospective design and its reflection of real-world clinical practice, providing evidence that may help optimize *H. pylori* eradication strategies and guidelines in Russia, a country with a relatively high infection rate in the adult population [44]. Our study represents a comprehensive analysis of the use of rebamipide, demonstrating significant clinical benefit for specific treatment regimens (PPI + C + A and PPI + C + A + B) in real-world clinical practice in terms of improved efficacy.

## 5. Conclusions

In this observational Russian registry cohort, rebamipide adjunctive therapy was associated with higher *H. pylori* eradication rates when prescribed with first-line empirical PPI + C + A therapy, exceeding the clinically acceptable 90% threshold and reaching 93%. Rebamipide-containing bismuth quadruple PPI + C + A + B therapy was also associated with higher treatment effectiveness, achieving a 97% eradication rate, with the highest rates observed when high-dose PPIs were used. Although AEs were more frequent in the rebamipide group, this did not negatively affect treatment adherence, and no treatment discontinuations were attributed to AEs. Given the non-randomized design and baseline differences between groups, these findings should be interpreted as real-world associations and should be confirmed in dedicated controlled studies.

## Figures and Tables

**Table 1:** Baseline Characteristics and Eradication Regimens Prescription in the Rebamipide and Control Groups.

Overall Population [with Rebamipide and Without Rebamipide, 2020–2024]		
Demographics (n = 4581)		
Age, Mean ± SD	48 ± 15	
Females, n (%)	2937 (64%)	
Males, n (%)	1644 (36%)	
Indication		
Dyspepsia, n (%)	3034 (66%)	
Peptic ulcer disease, n (%)	819 (18%)	
Duodenal, n (%)	395 (8.6%)	
Gastric, n (%)	424 (9.3%)	
Other, n (%)	727 (16%)	
	Population with Rebamipide (n = 820)	Population without Rebamipide (n = 3761)
Age, Mean ± SD	47 ± 15	49 ± 15
Females, n (%)	492 (60%) *	2445 (65%) *
Males, n (%)	328 (40%) *	1316 (35%) *
Indication		
Dyspepsia, n (%)	493 (60%) *	2541 (68%) *
Peptic ulcer disease, n (%)	220 (27%) *	599 (16%) *
Duodenal, n (%)	66 (8%)	329 (8.8%)
Gastric, n (%)	154 (19%) *	270 (7.2%) *
Other, n (%)	107 (13%) *	620 (16%) *
	Prescriptions (n, %) with Rebamipide	Prescriptions (n, %) (without Rebamipide)
Overall treatments	679 (83%) ^1^	3042 (81%) ^2^
PPI + C + A	168 (21%) *	453 (12%) *
PPI + M + Tc + B	6 (1%) *	244 (7%) *
PPI + C + A + B	486 (59%) *	2085 (55%) *
PPI + A + J + B	19 (2%) *	260 (7%) *

Abbreviations: PPI—proton pump inhibitor, C—clarithromycin, A—amoxicillin, M—metronidazole, Tc—tetracycline, B—bismuth salts, J—josamycin; PPI+C+A—triple eradication therapy combining a PPI with clarithromycin and amoxicillin; PPI + M + Tc + B—bismuth quadruple therapy combining a PPI, metronidazole, tetracycline, and bismuth; PPI + C + A + B—bismuth-based quadruple combining PPI with clarithromycin, amoxicillin, and bismuth; PPI + A + J + B—bismuth-based quadruple combining PPI with amoxicillin, josamycin and bismuth. * *p* < 0.05 in rebamipide group vs. without rebamipide ^1^. The % represents the number of patients treated with the below mentioned regimens with rebamipide among the overall patients treated with rebamipide-containing regimens. ^2^ The % represents the number of patients treated with the below mentioned regimens without rebamipide among the overall patients treated with regimens not containing rebamipide.

**Table 2:** Effectiveness of First-Line Regimens with and without Rebamipide, Depending on the Duration and PPI Dose.

mITT*, Eradication Rate, n/N (%)								
		PPI Dose						Total
		Low		Standard		High		
Eradication regimens with rebamipide		59/62 (95.2%)		468/495 (94.5%)		138/139 (99.3%)		665/696 (95.5%) *
Eradication regimens without rebamipide		307/344 (89.2%)		1924/2120 (90.8%)		551/578 (95.3%)		2782/3041 (91.5%) *
* *p* = 0.000156								
		Duration						Total
		7 days		10 days		14 days		
Eradication regimens with rebamipide		4/4 (100%)		22/23 (95.7%)		705/734 (96%)		731/761 (96.1%) *
Eradication regimens without rebamipide		16/20 (80%)		227/243 (93.4%)		2540/2778 (91.4%)		2783/3041 (91.5%) *
* *p* = 0.000008								
	mITT, eradication rate, n/N (%)							
	PPI + C + A	PPI + C + A	PPI + M + Tc + B	PPI + M + Tc + B	PPI + C + A + B	PPI + C + A + B	PPI + A + J + B	PPI + A + J + B
PPI dose	With rebamipide	Without rebamipide	With rebamipide	Without rebamipide	With rebamipide	Without rebamipide	With rebamipide	Without rebamipide
Low	10/12 (83.3%)	57/74 (77%)	1/1 (100%)	14/15 (93.3%)	37/38 (97.4%)	177/188 (94.1%)	1/1 (100%)	29/31 (93.5%)
Standard	88/96 (91.7%)	188/247 (76.1%)	3/3 (100%)	131/141 (92.9%)	300/312 (96.2%)	1239/1320 (93.9%)	8/8 (100%)	166/178 (93.3%)
High	22/22 (100%)	34/34 (100%)	2/2 (100%)	68/71 (95.8%)	83/83 (100%)	361/370 (97.6%)	10/10 (100%)	38/45 (84.4%)
Total	120/130 (92.3%) *	279/355 (78.6%) *	6/6 (100%) **	213/227 (93.8%) **	420/433 (97%) ***	1777/1878 (94.6%) ***	19/19 (100%) ****	233/254 (91.7%) ****
* *p* = 0.000266; ** *p* = 1.000000; *** *p* = 0.047821; **** *p* = 0.377038								
	PPI-C-A	PPI-C-A	PPI + M + Tc + B	PPI + M + Tc + B	PPI + C + A + B	PPI + C + A + B	PPI + A + J + B	PPI + A + J + B
Duration	With rebamipide	Without rebamipide	With rebamipide	Without rebamipide	With rebamipide	Without rebamipide	With rebamipide	Without rebamipide
7 days	0	4/5 (80%)	0	0		7/9 (77.8%)	0	0
10 days	12/12 (100%)	43/51 (84.3%)	0	5/5 (100%)	8/8 (100%)	131/136 (96.3%)	0	8/8 (93.1%)
14 days	137/147 (93.2%)	232/299 (77.6%)	6/6 (100%)	209/223 (93.7%)	449/462 (97.2%)	1636/1730 (94.6%)	19/19 (100%)	223/244 (91.4%)
Total	149/159 (93.7%) *	279/355 (78.6%) *	6/6 (100%) **	214/228 (93.9%) **	457/470 (97.2%) ***	1774 /1875 (94.6%) ***	19/19 (100%) ****	231/252 (91.7%) ****
* *p* = 0.000010; ** *p* = 1.000000; *** *p* = 0.016264; **** *p* = 0.377198								

*mITT (modified ITT)—includes all patients who completed follow-up observation (i.e., who underwent a confirmatory test after eradication therapy), regardless of adherence to protocol drug intake; PPI—proton pump inhibitor, C—clarithromycin, A—amoxicillin, M -metronidazole, Tc—tetracycline, B—bismuth salts, J—josamycin; PPI + C + A—triple regimen combining PPI with clarithromycin and amoxicillin; PPI + C + A + B—quadruple therapy combining PPI with clarithromycin, amoxicillin, and bismuth; PPI + A + B + J—quadruple therapy combining PPI with amoxicillin, josamycin and bismuth; PPI + M + Tc + B—bismuth quadruple therapy combining PPI, metronidazole, tetracycline, and bismuth. n—number of patients with effective eradication. N—number of patients with a particular eradication regimen.

**Table 3:** Safety of First-Line Treatments with and without Rebamipide, n/N (%).

	Dysgeusia n = 3472	Diarrhoea n = 3472	Nausea n = 3472	Vomiting n = 3472	Dyspepsia n = 3472	Heartburn n = 3472	Abdominal Pain n = 3472	Asthenia n = 3472	Anorexia n = 3472	Others n = 3472
Without rebamipide	145/3472 (4.2%)	257/3472 (7.4%) *	235/3472 (6.8%) *	63/3472 (1.8%) *	191/3472 (5.5%) *	74/3472 (2.1%)	97/3472 (2.8%) *	44/3472 (1.3%) *	8/3472 (0.2%)	156/3472 (4.5%)
With rebamipide	36/789 (4.6%)	40/789 (5.1%) *	114/789 (14.4%) *	48/789 (6.1%) *	105/789 (13.3%) *	19/789 (2.4%)	48/789 (6.1%) *	26/789 (3.3%) *	5/789 (0.6%)	31/789 (3.9%)

n—number of patients with adverse events. N—total number of patients. * *p* < 0.05 in rebamipide group vs. without rebamipide.

**Table 4:** Safety of Individual First-Line Treatments with and without Rebamipide, n/N (%).

Symptom	PPI + C + A	PPI + C + A	PPI + M + Tc + B	PPI + M + Tc + B	PPI + C + A + B	PPI + C + A + B	PPI + A + B + J	PPI + A + B + J
	With Rebamipide	Without Rebamipide	With Rebamipide	Without Rebamipide	With Rebamipide	Without Rebamipide	With Rebamipide	Without Rebamipide
Dysgeusia	5/149 (3.4%)	28/678 (4.1%)	0/3 (0%)	15/237 (6.3%)	18/410 (4.4%)	101/2255 (4.5%)	1/20 (5%)	7/286 (2.4%)
Diarrhea	8/149 (5.4%)	48/678 (6.2%)	0/3 (0%)	15/237 (6.3%)	10/410 (2.4%)	173/2255 (7.7%)	3/20 (15%)	37/286 (12.9%)
Nausea	15/149 (10.1%)	35/678 (5.2%)	0/3 (0%)	24/237 (10.1%)	55/410 (13.4%)	111/2255 (4.9%)	1/20 (5%)	14/286 (4.9%)
Vomiting	6/149 (4%)	13/678 (1.9%)	1/3 (33.3%)	4/237 (1.7%)	36/410 (8.8%)	52/2255 (2.3%)	1/20 (5%)	1/286 (0.3%)
Dyspepsia	15/149 (10.1%)	25/678 (3.7%)	1/3 (33.3%)	7/237 (3%)	84/410 (20.5%)	145/2255 (6.4%)	0/20 (0%)	7/286 (2.4%)
Heartburn	2/149 (1.3%)	5/678 (0.7%)	0/3 (0%)	1/237 (0.4%)	15/410 (3.7%)	64/2255 (2.8%)	0/20 (0%)	0/286 (0%)
Abd pain	7/149 (4.7%)	27/678 (4%)	0/3 (0%)	1/237 (0.4%)	34/410 (8.3%)	65/2255 (2.9%)	0/20 (0%)	14/286 (4.9%)
Asthenia	0/149 (0%)	5/678 (0.7%)	0/3 (0%)	1/237 (0.4%)	24/410 (5.9%)	51/2255 (2.3%)	0/20 (0%)	3/286 (1%)
Anorexia	0/149 (0%)	1/678 (0.1%)	0/3 (0%)	0/237 (0%)	3/410 (0.7%)	4/2255 (0.2%)	0/20 (0%)	1/286 (0.3%)
Others	7/149 (4.7%)	20/678 (2.9%)	0/3 (0%)	20/237 (8.4%)	19/410 (4.6%)	123/2255 (5.5%)	0/20 (0%)	16/286 (5.6%)
Adverse events (total)	52/149 (34.9%)	132/678 (19.5%)	2/3 (66.7%)	66/235 (28.1%)	223/410 (54.4%)	710/2246 (31.6%)	3/20 (15%)	65/286 (22.7%)

n—number of patients with adverse events. N—total number of patients.

**Table 5:** Compliance of First-Line Treatments with and without Rebamipide, n/N (%).

Overall 1st Line	Overall 1st Line	PPI-C-A	PPI-C-A	PPI + M + Tc + B	PPI + M + Tc + B	PPI + C + A + B	PPI + C + A + B	PPI + A + B + J	PPI + A + B + J
With Rebamipide	Without Rebamipide	With Rebamipide	Without Rebamipide	With Rebamipide	Without Rebamipide	With Rebamipide	Without Rebamipide	With Rebamipide	Without Rebamipide
764/773 (98.8%)	3314/3366 (98.5%)	163/164 (99.4%)	430/444 (96.8%)	5/6 (83.3%) *	230/234 (98.3%) *	469/474 (98.9%)	1983/2008 (98.8%)	18/19 (94.7%)	256/260 (98.5%)

n—number of patients with adverse events. N—total number of patients. * *p* < 0.05 in rebamipide group vs. without rebamipide.

## Data Availability

Raw data were generated at AEG-REDCap. De-identified raw data referring to the current study are available from the WorldHpReg and Hp-EuReg Scientific director and the PI of the project (OPN and JPG respectively) upon request. Individual participant data will not be shared.

## Abbreviations

The following abbreviations are used in this manuscript:

A	Amoxicillin
AE	Adverse Event
B	Bismuth Salts
C	Clarithromycin
Hp-EuReg	European Registry on *Helicobacter pylori* Management
*H. pylori*	*Helicobacter pylori*
ITT	Intention-to-Treat
J	Josamycin
M	Metronidazole
mITT	Modified Intention-to-Treat
N	Number of Cases
PPI	Proton Pump Inhibitor
RCT	Randomised Controlled Trial
SD	Standard Deviation
Tc	Tetracycline
AEG	Asociación Española de Gastroenterología
CI	Confidence interval
e-CRF	Electronic case report form
OR	Odds ratio
REDCap	Research Electronic Data Capture
WorldHpReg	World Registry on *H. pylori* Management
